# Niche-mediated depletion of the normal hematopoietic stem cell reservoir by Flt3-ITD–induced myeloproliferation

**DOI:** 10.1084/jem.20161418

**Published:** 2017-07-03

**Authors:** Adam J. Mead, Wen Hao Neo, Nikolaos Barkas, Sahoko Matsuoka, Alice Giustacchini, Raffaella Facchini, Supat Thongjuea, Lauren Jamieson, Christopher A.G. Booth, Nicholas Fordham, Cristina Di Genua, Deborah Atkinson, Onima Chowdhury, Emmanouela Repapi, Nicki Gray, Shabnam Kharazi, Sally-Ann Clark, Tiphaine Bouriez, Petter Woll, Toshio Suda, Claus Nerlov, Sten Eirik W. Jacobsen

**Affiliations:** 1 Haematopoietic Stem Cell Biology Laboratory, Weatherall Institute of Molecular Medicine, University of Oxford, Oxford, UK; 2 Medical Research Council Molecular Haematology Unit, Weatherall Institute of Molecular Medicine, University of Oxford, Oxford, UK; 3 Computational Biology Research Group, Weatherall Institute of Molecular Medicine, University of Oxford, Oxford, UK; 4 Department of Safety Research on Blood and Biological Products, National Institute of Infectious Diseases, Tokyo, Japan; 5 Department of Medicine Huddinge, Center for Hematology and Regenerative Medicine, Karolinska Institutet, Stockholm, Sweden; 6 Department of Cell and Molecular Biology, Wallenberg Institute for Regenerative Medicine, Karolinska Institutet, Stockholm, Sweden; 7 Cancer Science Institute, National University of Singapore, Singapore; 8 Karolinska University Hospital, Stockholm, Sweden

## Abstract

Although previous studies suggested that the expression of FMS-like tyrosine kinase 3 (Flt3) initiates downstream of mouse hematopoietic stem cells (HSCs), *FLT3* internal tandem duplications (*FLT3* ITDs) have recently been suggested to intrinsically suppress HSCs. Herein, single-cell interrogation found *Flt3* mRNA expression to be absent in the large majority of phenotypic HSCs, with a strong negative correlation between *Flt3* and HSC-associated gene expression. Flt3-ITD knock-in mice showed reduced numbers of phenotypic HSCs, with an even more severe loss of long-term repopulating HSCs, likely reflecting the presence of non-HSCs within the phenotypic HSC compartment. Competitive transplantation experiments established that Flt3-ITD compromises HSCs through an extrinsically mediated mechanism of disrupting HSC-supporting bone marrow stromal cells, with reduced numbers of endothelial and mesenchymal stromal cells showing increased inflammation-associated gene expression. Tumor necrosis factor (TNF), a cell-extrinsic potent negative regulator of HSCs, was overexpressed in bone marrow niche cells from FLT3-ITD mice, and anti-TNF treatment partially rescued the HSC phenotype. These findings, which establish that Flt3-ITD–driven myeloproliferation results in cell-extrinsic suppression of the normal HSC reservoir, are of relevance for several aspects of acute myeloid leukemia biology.

## Abstract (precis)

Flt3 expression is absent in the large majority of phenotypic hematopoietic stem cells (HSCs). Mead et al. show that FLT3-ITD–driven myeloproliferation causes cell-extrinsic suppression of the normal HSC reservoir through disruption of HSC-supporting BM stromal cells, including overexpression of TNF.

## Introduction

Suppression and collapse of normal blood cell replenishment underlies the severe morbidity and high mortality accompanying many hematologic malignancies, including acute myeloid leukemia (AML; Löwenberg et al., 1999). However, the intrinsic and extrinsic cellular and molecular mechanisms responsible for this suppression of normal hematopoiesis remain to be established and are of critical importance toward development of improved and targeted therapies.

Studies of the surface expression and function of FMS-like tyrosine kinase 3 (Flt3) have provided fundamental insights into its role in normal hematopoiesis (Luc et al., 2007; Boyer et al., 2012). Within the hematopoietic progenitor compartment, expression of Flt3 is present in multipotent progenitors (MPPs), including lymphoid-primed MPPs (LMPPs; Adolfsson et al., 2001, 2005; Boyer et al., 2011; Buza-Vidas et al., 2011) and early lymphoid (Luc et al., 2012) and myeloid (Böiers et al., 2010) progenitor populations. Genetic knockout approaches established an important role for Flt3 in the maintenance of these early progenitor cell populations (Sitnicka et al., 2002; Böiers et al., 2010). In contrast, up-regulation of surface expression of Flt3 marks loss of self-renewal of mouse hematopoietic stem cells (HSCs; Adolfsson et al., 2001; Christensen and Weissman, 2001), and importantly, genetic knockout studies of *Flt3*, or its ligand, have demonstrated no significant impact of loss of Flt3 on the normal HSC compartment (Buza-Vidas et al., 2009). Moreover, lineage-tracing experiments demonstrated that although *Flt3* transcriptional expression first occurs in a minor population of cells within the phenotypic HSC compartment, these cells in fact lack self-renewal capability and therefore represent progenitors rather than bona fide HSCs (Boyer et al., 2011, 2012; Buza-Vidas et al., 2011). Those results highlight the well-recognized heterogeneity of the phenotypic HSC compartment, which in addition to genuine HSCs contains non-HSC progenitors, marked in part by expression of *Flt3* transcript (Purton and Scadden, 2007; Boyer et al., 2011; Buza-Vidas et al., 2011).

Constitutively activating internal tandem duplications (ITDs) of *FLT3* are one of the most common, recurrent somatic mutations found in patients with AML (Meshinchi and Appelbaum, 2009). Although *FLT3* ITDs often occur as a secondary mutation (Gale et al., 2008), there are also cases in which they clearly originate in the founding leukemic clone (Ding et al., 2012), and it is clear that *FLT3* ITDs act as a potent driver mutation (Smith et al., 2012) and confer a poor outcome because of high relapse risk (Gale et al., 2008). Thus, it is of considerable importance to understand which cells propagate FLT3-ITD–associated myeloid disease and how these cells contribute to clonal dominance over normal hematopoietic cells to result in the hematopoietic suppression typically observed in patients (Löwenberg et al., 1999). Although *FLT3-ITD* mutations are present in the primitive human CD34^+^CD38^−^ stem/progenitor cell compartment, including LMPP-like cells (Levis et al., 2005; Goardon et al., 2011; Mead et al., 2013), *FLT3* ITDs appear to be absent in the majority of “preleukemic” HSCs in patients with FLT3-ITD AML (Jan et al., 2012). However, in a recent study of Flt3-ITD knock-in mice in which phenotypic HSCs were reduced, *Flt3* mRNA was found to be expressed in the phenotypically defined HSC compartment when analyzed at the cell population level (Chu et al., 2012), and based on this and other findings, it was concluded that a HSC-intrinsic mechanism is responsible for the observed HSC suppression in Flt3-ITD mice (Chu et al., 2012). Importantly, this implicates a previously unrecognized HSC-intrinsic role for Flt3 and Flt3-ITD in governing the dynamics of the HSC compartment and potentially radically revises our understanding of the role of Flt3 in normal hematopoiesis and the impact of FLT3-ITDs on HSCs in hematologic malignancies.

Because a definitive description of the heterogeneous expression pattern of Flt3 is important for understanding the physiological role of the receptor and the potential intrinsic impact of Flt3-ITD in HSC homeostasis, we herein explored *Flt3* expression at the transcriptional and protein levels in the phenotypically defined HSC compartment at the single-cell level. We then explored whether the observed HSC suppression in Flt3-ITD mice might rather involve an HSC-extrinsic mechanism, including an impact on components of the HSC niche.

## Results

### *Flt3* mRNA is expressed only in a small and distinct subset of phenotypically defined mouse HSCs

**Figure 1.**
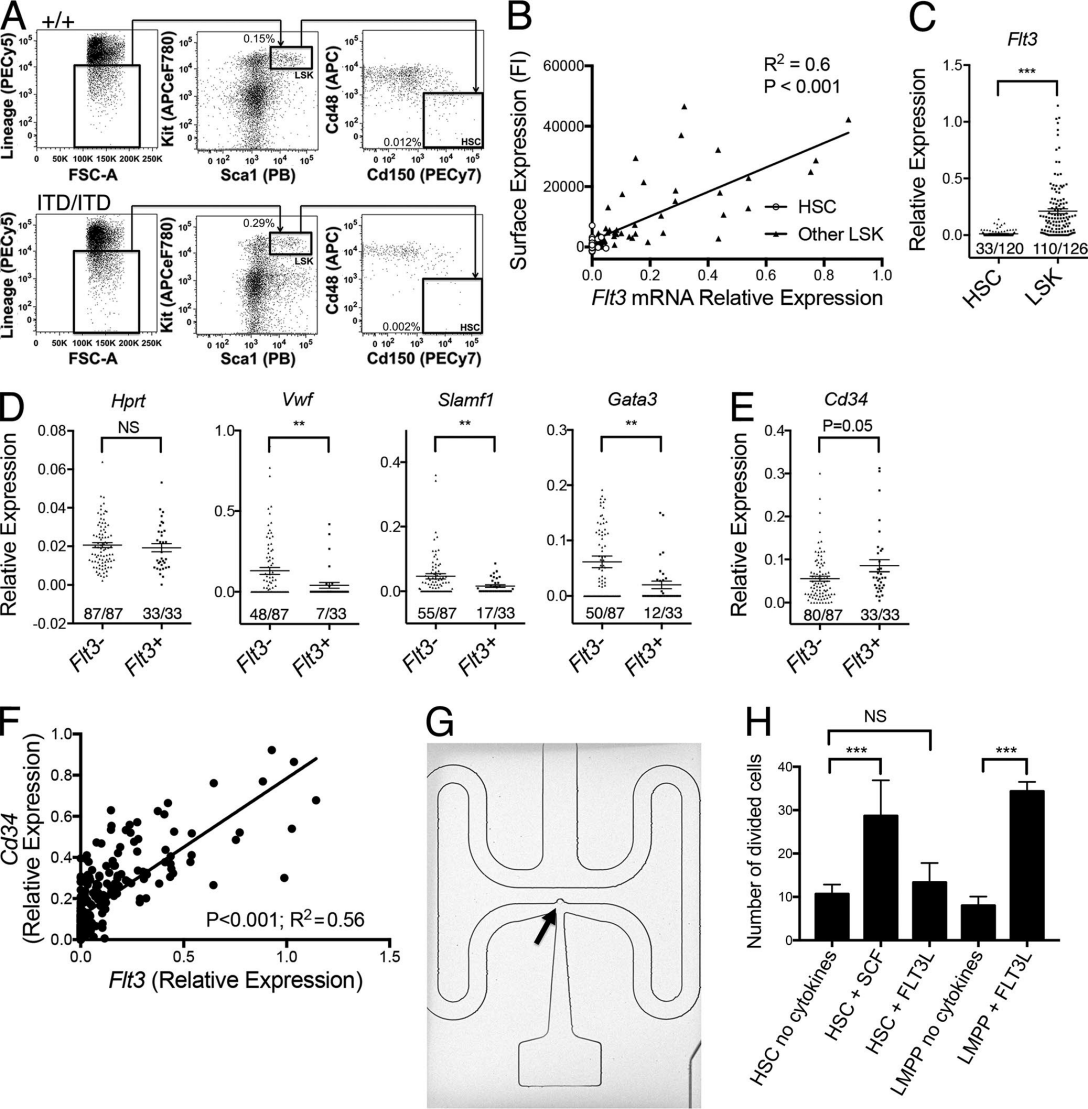
**Single-cell analysis of the phenotypic HSC compartment.** (A) Representative flow cytometry–based analysis and quantification of LSK cells and phenotypic (LSKCD150^+^CD48^−^) HSCs in 8–10-wk-old *Flt3^+/+^* and *Flt3^ITD/ITD^* mice. Shown are the mean values for the frequencies (percentage of total BM cells) of the indicated population across all experiments (+/+, *n* = 31 mice; ITD/ITD, *n* = 23 mice). (B) Correlation of Flt3 protein cell-surface expression (fluorescence intensity; FI) and *Flt3* relative mRNA gene expression level in 28 phenotypic (LSKCD150^+^48^−^) HSCs (open circles) and 62 single non-HSC LSK cells (filled triangles) from two 8-wk-old *Flt3^+/+^* mice. P-value represents Pearson correlation coefficient. (C) *Flt3* relative mRNA gene expression level in 120 LSKCD150^+^48^−^ HSCs and 126 single non-HSC LSK cells from 8-wk-old *Flt3^+/+^* mice (*n* = 5 mice). P-value at the top of the graph represents unpaired *t* test; the *x*/*y* figures beneath each dot plot indicate the number of cells demonstrating amplification for indicated gene (*x*) and total numbers of cells analyzed (*y*). (D) Relative mRNA gene expression level of *Hprt*, *Vwf*, *Slamf1*, and *Gata3* in 120 LSKCD150^+^48^−^ HSCs from 8-wk-old *Flt3^+/+^* mice stratified according to presence (*n* = 33) or absence (*n* = 87) of *Flt3* expression (*n* = 5 mice). (E) Relative mRNA gene expression level of *Cd34* in 120 LSKCD150^+^48^−^ HSCs from 8-wk-old *Flt3^+/+^* mice stratified according to presence (*n* = 33) or absence (*n* = 87) of *Flt3* expression (*n* = 5 mice). (F) Correlation of *Flt3* and *Cd34* relative mRNA gene expression level in 120 phenotypic HSCs and 126 single non-HSC LSKs from 8-wk-old *Flt3^+/+^* mice (*n* = 5 mice). (G) Single mouse phenotypic HSC (arrow) captured in a 4.5-nl reaction chamber for single-cell gene expression analysis (representative image from one experiment). (H) Number of cells undergoing cell division after incubation of 180 single LSKCD150^+^48^−^ HSCs or LSKFlt3^high^ LMPPs under the indicated conditions for 5 d followed by culture in a full cytokine cocktail (see Materials and Methods) for a further 10 d. SCF indicates stem cell factor, and FLT3L indicates FLT3 ligand. Three independent experiments with 60 cells per experiment. P-values represent χ^2^ analysis comparing frequency of cells undergoing cell division of a total of 180 plated cells in the three experiments. Data are shown as mean (SEM) values. **, P < 0.01; ***, P < 0.001. NS, not significant.

We first sought to investigate heterogeneity of Flt3 expression in phenotypically defined HSCs, by determining the relationship between *Flt3* mRNA and cell-surface Flt3 protein expression at the single-cell level. Index FACS of 90 WT (*Flt3^+/+^*) Lin^−^Sca1^+^Kit^+^ (LSK) cells (Fig. 1 A), encompassing all HSCs as well as distinct subpopulations of MPPs, showed a significant correlation between surface Flt3 protein and *Flt3* mRNA expression in the same single cells (Fig. 1 B; P < 0.001). All 28 LSK cells within the stringent phenotypic HSC compartment (LSKCD150^+^48^−^) clearly showed much lower expression of both Flt3 protein and *Flt3* mRNA than other LSK cells (Fig. 1 C). Gene expression analysis of 120 single LSKCD150^+^48^−^ cells and 126 single LSK cells not residing in the LSKCD150^+^48^−^ HSC compartment showed that as many as 72% of LSKCD150^+^48^−^ cells did not express any detectable *Flt3* at the single-cell level as opposed to only 13% of other (MPP) LSK cells (Fig. 1 C; P < 0.001). Moreover, the minority of phenotypic HSCs expressing *Flt3* mRNA did so at a much lower level than did other (MPP) LSK cells (Fig. 1 C; P < 0.001). Single-cell gene expression analysis of HSCs stratified according to presence (*n* = 33) or absence (*n* = 87) of *Flt3* expression revealed reduced frequencies and/or levels of expression of key stem cell–associated genes (*Vwf*, *Slamf1*, and *Gata3;* Fig. 1 D) in *Flt3* mRNA–positive cells. Indeed, only 7 of 55 (13%) *Vwf*-expressing LSKCD150^+^48^−^ HSCs, which reside at the apex of the hematopoietic hierarchy (Sanjuan-Pla et al., 2013), showed detectable *Flt3* mRNA expression. Further, *Flt3* mRNA–negative HSCs showed reduced *Cd34* mRNA expression, characteristic of adult mouse HSCs (Osawa et al., 1996; P = 0.05; Fig. 1 E), with strong correlation between *Flt3* and *Cd34* mRNA expression in LSK cells (P < 0.001, Fig. 1 F). Together, these findings support that within the phenotypic HSC compartment, expression of *Flt3* mRNA correlates with loss of HSC-associated gene expression programs. Importantly, gene expression of single LSKCD150^+^48^−^ cells captured in nanofluidic reaction chambers (Fig. 1 G), with sensitivity for gene expression detection to the single-molecule level (Wu et al., 2014), showed that 12 of 17 (71%) captured LSKCD150^+^48^−^ cells did not express any *Flt3* mRNA, supporting that the absence of *Flt3* expression is unlikely to represent a lack of sensitivity of the assay.

To confirm that LSKCD150^+^48^−^ cells were not functionally responsive to FLT3 ligand (FLT3L), we cultured single LSKCD150^+^48^−^ cells in the absence of cytokines or in the presence of FLT3L or stem cell factor (SCF), the ligand for c-kit shown to be highly expressed on HSCs (Ikuta and Weissman, 1992). Importantly, both of these cytokines have been shown to act as potent viability factors for stem and progenitor cells expressing the corresponding receptors (Veiby et al., 1996). After a 5-d preincubation period, we added a strongly proliferative cytokine cocktail to promote the proliferation of cells that had survived the preincubation period, thereby assessing the ability of SCF and FLT3L to act as viability factors for LSKCD150^+^48^−^-enriched HSCs. In agreement with previous studies (Yang et al., 2005), SCF markedly promoted the survival of LSKCD150^+^48^−^-enriched HSCs (Fig. 1 H). In marked contrast, FLT3L had no ability to enhance survival of LSKCD150^+^48^−^ cells, although in agreement with previous studies (Adolfsson et al., 2001), it potently enhanced survival of LSKFlt3^high^ LMPPs (Fig. 1 H). Together, these data along with previous studies (Adolfsson et al., 2001; Christensen and Weissman, 2001; Boyer et al., 2011, 2012; Buza-Vidas et al., 2011), provide several lines of evidence supporting that Flt3 is not expressed at functionally relevant levels in bona fide mouse HSCs.

### Flt3-ITD–induced myeloproliferation results in a progressive loss of *Flt3* mRNA–negative HSCs

**Figure 2.**
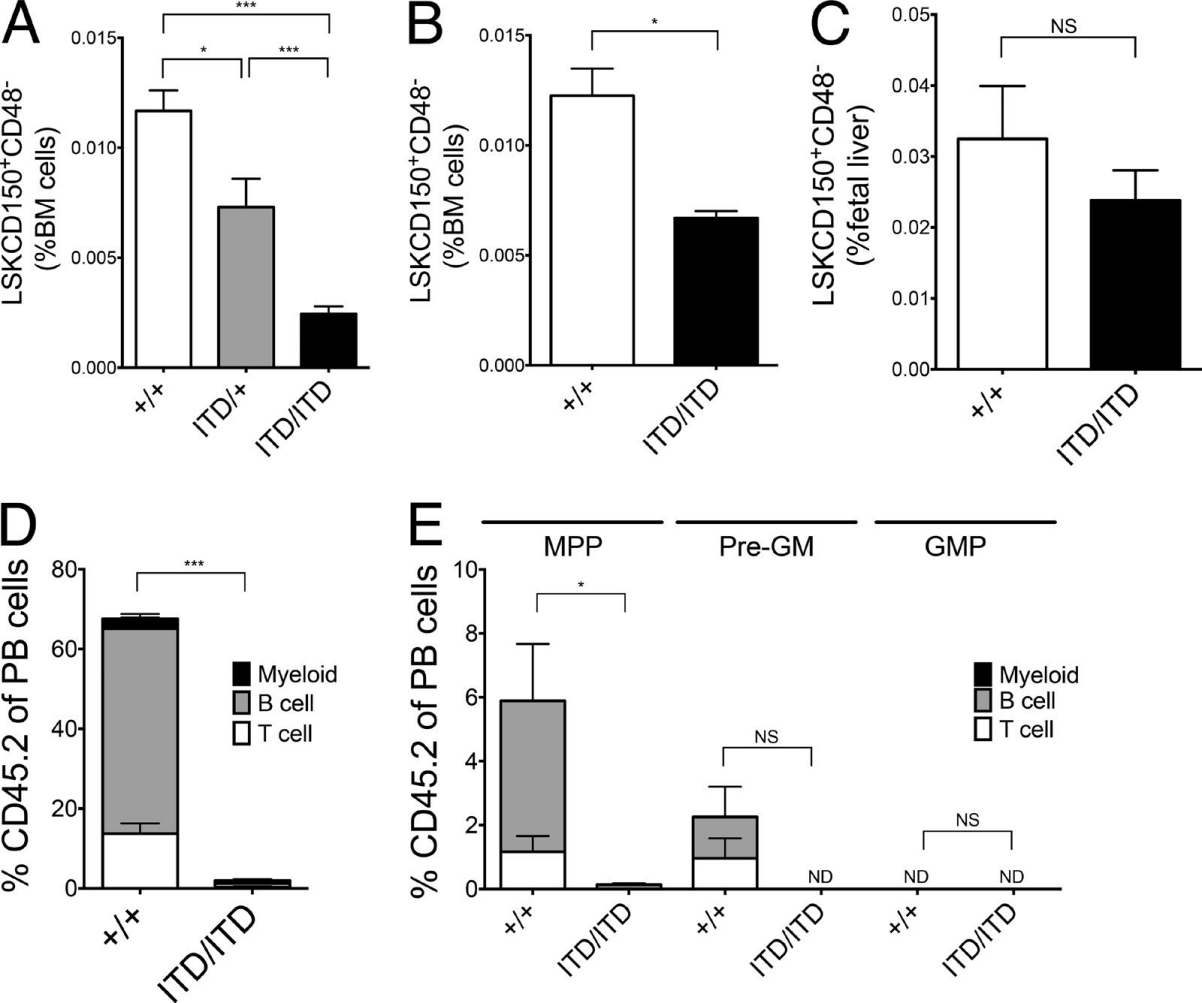
**Flt3-ITDs cause gene dosage–dependent and progressive suppression of BM HSCs.** (A) Gene dosage–dependent reduction in LSKCD150^+^48^−^ cells in 8–10-wk-old *Flt3^+/+^*, *Flt3^ITD/+^*, and *Flt3^ITD/ITD^* mice (+/+, *n* = 31; ITD/+, *n* = 14; ITD/ITD, *n* = 23). (B) Reduction in LSKCD150^+^48^−^ cells in 2-wk-old *Flt3^ITD/ITD^* mice (+/+, *n* = 4; ITD/ITD, *n* = 6). (C) Numbers of phenotypic (LSKCD150^+^CD48^−^) HSCs in E15 FL from *Flt3^+/+^* and *Flt3^ITD/ITD^* mice (+/+, *n* = 8; ITD/ITD, *n* = 9). (D) Percentage CD45.2 chimerism in peripheral blood of recipient mice 16 wk after transplantation of 500,000 unfractionated CD45.2 BM cells and 200,000 CD45.1 WT competitor cells (eight to nine recipients per genotype in three experiments). (E) Percentage CD45.2 chimerism in BM of recipient (CD45.1) mice 16 wk after transplantation of 5,000 CD45.2 MPPs (LSKCD150^−^CD48^+^), pre-GM (Lin^−^Kit^+^Sca1^−^CD41^−^CD16/32^−^CD150^−^CD105^−^), and GMPs (Lin^−^Kit^+^Sca1^−^CD41^−^CD16/32^+^) together with 200,000 CD45.1 WT BM competitor cells (eight to nine recipients per genotype in two experiments). Data are shown as mean (SEM) values. *, P < 0.05; ***, P < 0.001 by *t* test. NS, not significant.

Despite the expansion of LSK cells in Flt3-ITD mice (Lee et al., 2007; Kharazi et al., 2011), there was a gene dosage–dependent reduction of LSKCD150^+^48^−^ phenotypic HSCs in the BM of Flt3-ITD mice (Fig. 2 A; P < 0.001) that paralleled the increased severity of myeloproliferation in homozygous mice (Kharazi et al., 2011). The loss of phenotypic HSCs in *Flt3^ITD/ITD^* mice occurred progressively and in parallel to the development of myeloproliferation (Lee et al., 2007), with a mild HSC phenotype already apparent in 2-wk-old mice (Fig. 2 B); notably, LSKCD150^+^48^−^ cells were present at normal numbers in Flt3-ITD fetal livers (FLs) before the development of overt myeloid disease (Fig. 2 C; Mead et al., 2013). Transplantation of 500,000 BM cells from 8–10-wk-old *Flt3^+/+^* or *Flt3^ITD/ITD^* (CD45.2) mice together with 200,000 WT (CD45.1) BM cells showed a profound loss of functional/repopulating HSCs as defined by multilineage reconstitution (Fig. 2 D). Because 500,000 *Flt3^ITD/ITD^* BM cells will include 10–15 phenotypic HSCs for each recipient mouse, the failure to observe significant engraftment in any recipient mouse (*n* = 15) was surprising. Furthermore, transplantation of large numbers of cells purified from the expanded progenitor populations also failed to sustain long-term *Flt3^ITD/ITD^* myeloproliferation (Fig. 2 E). Together, these data support that the BM of *Flt3^ITD/ITD^* mice contains very few long-term repopulating HSCs.

**Figure 3.**
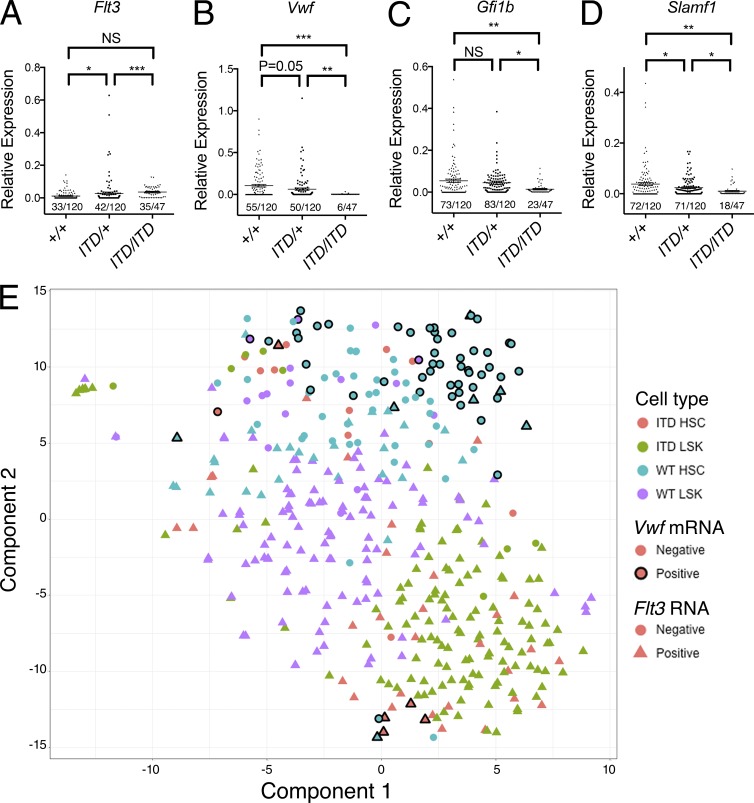
**The cellular composition of the phenotypic HSC compartment is severely disrupted in Flt3-ITD mice.** (A–D) Relative mRNA gene expression level for *Flt3* (A), *Vwf* (B), *Gfi1b* (C), and *Slamf1* (D) in LSKCD150^+^48^−^ single cells from 8-wk-old *Flt3^+/+^* (*n* = 120), *Flt3^ITD/+^* (*n* = 120), and *Flt3^ITD/ITD^* (*n* = 47) mice (*n* = 3–5 mice for each genotype). The *x*/*y* figures beneath each dot plot indicate the number of cells demonstrating amplification for indicated gene (*x*) and total numbers of cells analyzed (*y*). (E) t-SNE analysis demonstrating distinct clustering of HSCs and total LSK cells from *Flt3^+/+^* and *Flt3^ITD/ITD^* mice. Color of each point indicates cell type: red, *Flt3^ITD/ITD^* HSC (*n* = 47); green, *Flt3^ITD/ITD^* LSK (*n* = 133); blue, *Flt3^+/+^* HSC (*n* = 120); and purple, *Flt3^+/+^* LSK (*n* = 126). Presence (triangles) or absence (circles) of *Flt3* mRNA and presence (black border) or absence (no border) of *Vwf* mRNA is also indicated. The large majority of phenotypic HSCs from *Flt3^ITD/ITD^* mice cluster together with LSK progenitor cells and are clearly separated from *Vwf* mRNA–positive *Flt3^+/+^* HSCs. Data are shown as mean (SEM) values. *, P < 0.05; **, P < 0.01; ***, P < 0.001 by *t* test. NS, not significant.

To explore the discrepancy between phenotypic (LSKCD150^+^48^−^) and long-term repopulating HSCs in *Flt3^ITD/ITD^* mice, we performed single-cell gene expression analysis on the cells residing in the phenotypic HSC compartment in Flt3-ITD mice. This analysis showed increased frequency of *Flt3* expression in single LSKCD150^+^48^−^ cells in Flt3-ITD mice, with 28, 35, and 74% of cells showing *Flt3* expression in *Flt3^+/+^*, *Flt3^ITD/+^*, and *Flt3^ITD/ITD^* mice, respectively (P < 0.001, Fig. 3 A), in keeping with a relative preservation (less than twofold reduction) of Flt3-ITD–expressing LSKCD150^+^48^−^ cells in *Flt3^ITD/ITD^* mice, as opposed to a 13-fold reduction of *Flt3* mRNA-negative LSKCD150^+^48^−^ cells. The disrupted composition of the HSC compartment in *Flt3^ITD/ITD^* mice was accompanied by reduced frequencies and levels of expression of the HSC-affiliated genes *Vwf, Gfi1b*, and *Slamf1* in *Flt3^ITD/+^* and *Flt3^ITD/ITD^* HSCs (Fig. 3, B–D), consistent with a 17-fold reduction of *Vwf* mRNA–positive HSCs in *Flt3^ITD/ITD^* mice, despite 87% of these cells not expressing any Flt3. Furthermore, *t*-distributed stochastic neighbor embedding (t-SNE) analysis clearly demonstrated that most cells residing in the phenotypic HSC compartment in *Flt3^ITD/ITD^* mice clustered more closely with LSK progenitor cells than with HSCs (Fig. 3 E). To confirm the distinct clustering of HSCs from *Flt3^ITD/ITD^* mice, we performed hierarchical clustering analysis. After excluding a small number of cells falling into a minor cluster, this analysis identified two major clusters of cells. The first HSC cluster contained 98 of 119 (82%) phenotypic WT HSCs but only 12 of 47 (26%) phenotypic *Flt3^ITD/ITD^* HSCs (P < 0.001). The LSK cluster contained 216 of 250 (86%) *Flt3^+/+^* and *Flt3^ITD/ITD^* LSKs. Crucially, 35 of 47 (74%) phenotypic *Flt3^ITD/ITD^* HSCs were contained within this LSK cluster versus only 21 of 119 (18%) *Flt3^+/+^* HSCs (P < 0.001).

Together, these data demonstrate a selective and profound depletion of *Vwf-*positive and *Flt3* mRNA–negative LSKCD150^+^48^−^ cells (likely to represent bona fide functional HSCs) and relative preservation of *Flt3*-expressing LSKCD150^+^48^−^ cells with reduced expression of HSC-affiliated genes (likely to represent contaminating progenitor cells). These data are in keeping with the LSKCD150^+^48^−^ compartment in *Flt3^ITD/ITD^* mice being primarily composed of “contaminating” *Flt3-*expressing progenitor cells rather than HSCs, helping to explain the failure to observe any long-term engraftment after transplantation of *Flt3^ITD/ITD^* BM despite only a moderate reduction in the LSKCD150^+^48^−^ compartment.

### The loss of HSCs in *Flt3^ITD/ITD^* mice involves cell-extrinsic mechanisms

**Figure 4.**
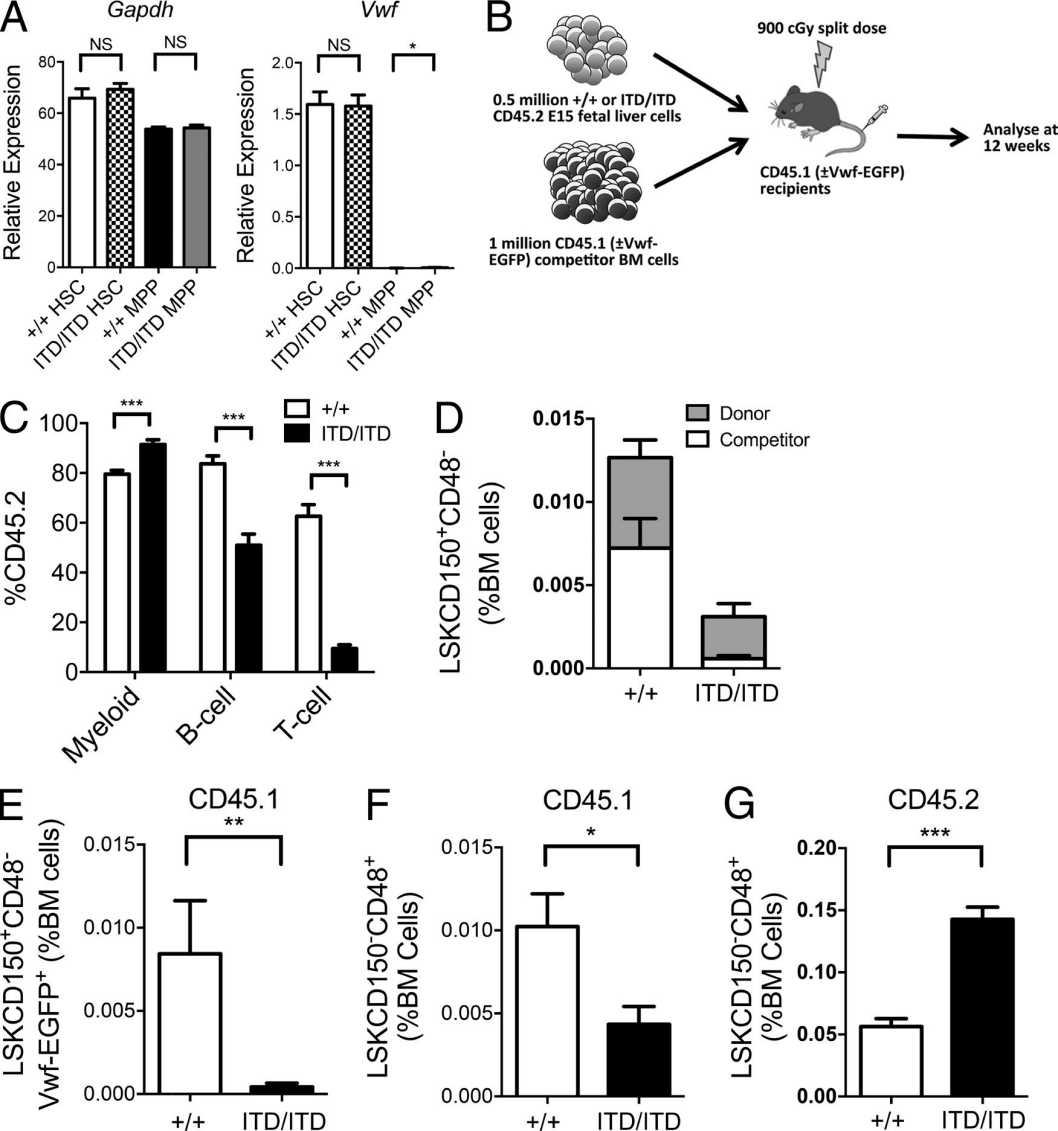
**Flt3-ITDs cause cell-extrinsic suppression of BM HSCs.** (A) Relative mRNA gene expression (normalized to *Hprt*) of *Gapdh* and *Vwf* in E15 fetal liver phenotypic HSCs (LSKCD150^+^CD48^−^) and MPPs (LSKCD150^−^CD48^+^) from Flt3^+/+^ and Flt3^ITD/ITD^ mice (*n* = 9 replicates from three mice per genotype). (B) Experimental design for competitive transplantation experiments. (C) Analysis of engraftment 12 wk after transplantation of 5 × 10^5^ CD45.2 E15 FL cells (Flt3^+/+^ or Flt3^ITD/ITD^) into CD45.1 recipients with 10^6^ WT CD45.1 BM competitor cells (four donor FL of each genotype transplanted into two to three recipients in two experiments). Results are expressed as percentage of cells expressing CD45.2 of each lineage, myeloid (Mac1^+^), B cell (B220^+^CD19^+^), and T cell (CD4^+^ and/or CD8^+^), in the peripheral blood 12 wk after transplantation. (D) Percentage (of total BM cells) of donor (CD45.2) and competitor (CD45.1) HSCs (LSKCD150^+^CD48^−^) in BM of recipient mice 12 wk after transplantation (four donor FL of each genotype transplanted into two to three recipients in two experiments). (E) Percentage competitor Vwf-EGFP^+^ HSCs (LSKCD150^+^CD48^−^Vwf-EGFP^+^CD45.1) in BM of recipient mice 12 wk after transplantation, expressed as percentage of total BM cells. (F and G) Percentage (of total BM cells) of competitor (CD45.1; F) or donor (CD45.2; G) MPPs (LSKCD150^−^CD48^+^) in BM of recipient mice 12 wk after transplantation (four donor FL of each genotype each transplanted into two to three recipients in two experiments). Data are shown as mean (SEM) values. *, P < 0.05; **, P < 0.01; ***, P < 0.001 by *t* test. NS, not significant.

The lack of *Flt3* mRNA expression in the majority of phenotypic HSCs is inconsistent with the proposed cell-intrinsic role for aberrant Flt3 signaling as an explanation for the marked suppression of the HSC compartment in *Flt3^ITD/ITD^* mice (Chu et al., 2012). The dramatic reduction of non–*Flt3*-expressing LSKCD150^+^48^−^ HSCs in Flt3-ITD mice appeared more compatible with the observed HSC suppression being mediated through an HSC-extrinsic mechanism. To explore this, we performed competitive transplantation experiments using Flt3-ITD FL cells (CD45.2), because they contain normal numbers of LSKCD150^+^48^−^ cells (Fig. 2 C) expressing normal levels of *Vwf* (Fig. 4 A), in keeping with an intact HSC compartment. Importantly, WT BM competitor (CD45.1) cells were cotransplanted to determine the extrinsic impact of Flt3-ITD on WT HSCs (Fig. 4 B). Unlike *Flt3^ITD/ITD^* BM cells, *Flt3^ITD/ITD^* FL cells showed robust engraftment and recapitulated Flt3-ITD–induced myeloproliferation (Fig. 4 C). *Flt3^ITD/ITD^* donor cells dominated hematopoiesis, with increased myeloid engraftment in comparison with WT FL transplanted mice (P < 0.001; Fig. 4 C), but reduced lymphoid engraftment (P < 0.001; Fig. 4 C) in keeping with the myeloid bias of *Flt3^ITD/ITD^* MPPs (Mead et al., 2013). Most notably, not only *Flt3^ITD/ITD^* donor but also WT competitor LSKCD150^+^48^−^ HSC-enriched cells were markedly reduced in recipient mice (Fig. 4 D), conclusively demonstrating that Flt3-ITD–expressing cells exert an extensive extrinsic suppression of the WT LSKCD150^+^48^−^ HSC compartment. In fact, the observed reduction of LSKCD150^+^48^−^ cells was more extensive in the WT (CD45.1) competitor BM than in the *Flt3^ITD/ITD^* donor cells, including vWF-EGFP–positive competitor HSCs (20-fold reduction; P < 0.01; Fig. 4 E), compatible with the observed HSC suppression being entirely HSC-extrinsically mediated. Also, competitor WT LSKCD150^−^48^+^ MPPs were reduced in *Flt3^ITD/ITD^*-engrafted mice, although less than HSCs (Fig. 4 F); in striking contrast, *Flt3^ITD/ITD^*-expressing MPPs (expressing high levels of Flt3) were markedly expanded (Fig. 4 G), suggesting that a cell-extrinsic negative impact of *Flt3^ITD/ITD^* selectively affects normal HSCs, and that *Flt3^ITD/ITD^* cell-intrinsically drives an expansion of LSKCD150^−^48^+^ MPPs.

### Flt3-ITD–induced myeloproliferation disrupts BM stromal cells

**Figure 5.**
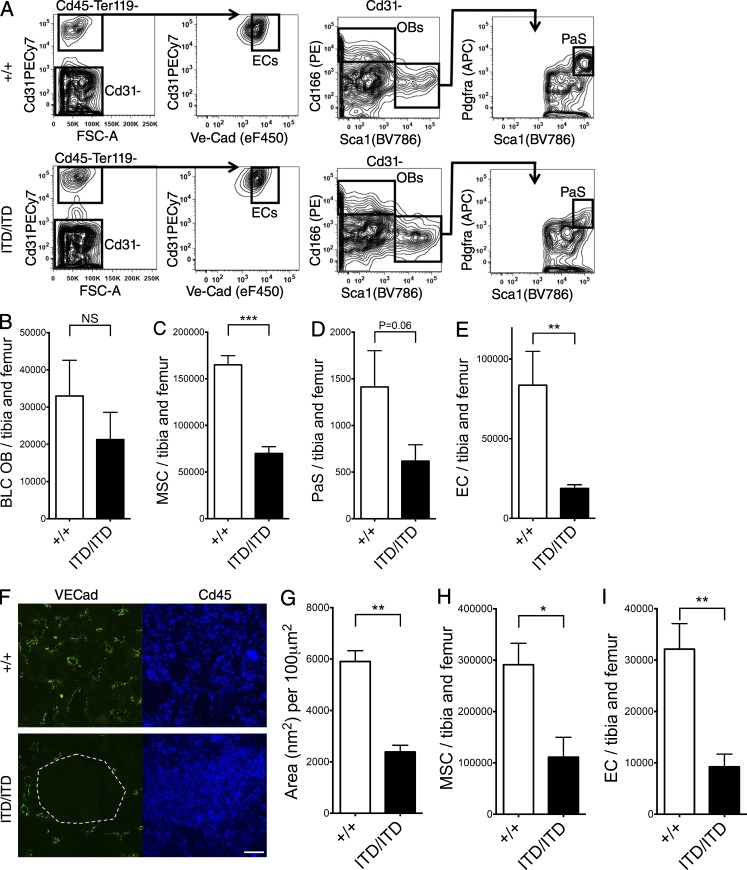
**Flt3-ITD–induced myeloproliferation disrupts the vascular HSC niche.** (A) Representative gating strategy to identify osteoblasts (OBs; CD45^−^Ter119^−^CD31^−^CD166^+^Sca1^−^), PaS MSCs (CD45^−^Ter119^−^CD31^−^CD166^−^Sca1^+^Pdgfra^+^), and ECs (CD45^−^Ter119^−^CD31^+^VE-Cadherin^+^) in BLCs from *Flt3^+/+^* and *Flt3^ITD/ITD^* mice. (B–E) FACS quantification of OBs in the BLC of 8–10-wk-old *Flt3^+/+^* and *Flt3^ITD/ITD^* mice (11 and 13 mice per genotype, respectively; B), MSCs (C) and PaS MSCs (D) in the BLC, and ECs in the BLC and BM (E) of 8–10-wk-old *Flt3^+/+^* and *Flt3^ITD/ITD^* mice (six and seven mice per genotype, respectively). (F) Representative images of *Flt3^+/+^* and *Flt3^ITD/ITD^* BM in which ECs are identified by VE-cadherin and hematopoietic cells by CD45 staining. An area devoid of vasculature and entirely replaced by CD45^+^ *Flt3^ITD/ITD^* hematopoietic cells is highlighted by the white dashed line. Bar, 50 µm. (G) Sum of blood vessel areas in *Flt3^+/+^* and *Flt3^ITD/ITD^* mouse BM (*n* = 15 areas from three mice of each genotype). (H and I) Numbers of MSCs (H) and ECs (I) in recipient mice 12 wk after transplantation of 5 × 10^5^ CD45.2 E15 FL cells (Flt3^+/+^ or Flt3^ITD/ITD^) into CD45.1 WT recipients with 10^6^ WT CD45.1 BM competitor cells (two donor FL of each genotype transplanted into two to three recipients in two experiments). Data are shown as mean (SEM) values. *, P < 0.05; **, P < 0.01; ***, P < 0.001 by *t* test. NS, not significant.

In view of the marked cell-extrinsic suppression of HSCs observed in *Flt3^ITD/ITD^* mice, we next analyzed key cellular components of the BM HSC niche to explore whether the nonhematopoietic BM microenvironment might be disrupted in *Flt3^ITD/ITD^* mice. FACS analysis of bone lining cells (BLCs; Fig. 5 A; Nakamura et al., 2010) revealed that numbers of BLC osteoblasts (CD45^−^Ter119^−^CD31^−^CD166^+^Sca1^−^; Fig. 5 B) were relatively normal. In contrast, total BM mesenchymal stromal cells (MSCs; CD45^−^Ter119^−^CD31^−^Pdgfra^+^; ×0.4; P < 0.001; Fig. 5 C), BLC Pdgfra^+^Sca1^+^ (Houlihan et al., 2012) MSCs (PaS; ×0.4; P = 0.06; Fig. 5 D), and total BM endothelial cells (ECs; CD45^−^Ter119^−^CD31^+^VE-cadherin^+^; ×0.21; P < 0.01; Fig. 5 E) were all reduced in number in the BM of *Flt3^ITD/ITD^* mice. Immunohistochemistry confirmed loss of ECs in the BM of *Flt3^ITD/ITD^* mice (P < 0.01; Fig. 5, F and G). To confirm that the observed changes in the niche were also present in transplanted WT recipient mice, CD45.1 mice were analyzed 12 wk after transplantation of 5 × 10^5^ CD45.2 E15 FL cells (*Flt3^+/+^* or *Flt3^ITD/ITD^*) with 10^6^ WT CD45.1 BM competitor cells. This analysis confirmed a similar reduction of MSCs (×0.38, P < 0.05; Fig. 5 H) and ECs (×0.29, P < 0.01; Fig. 5 I).

**Figure 6.**
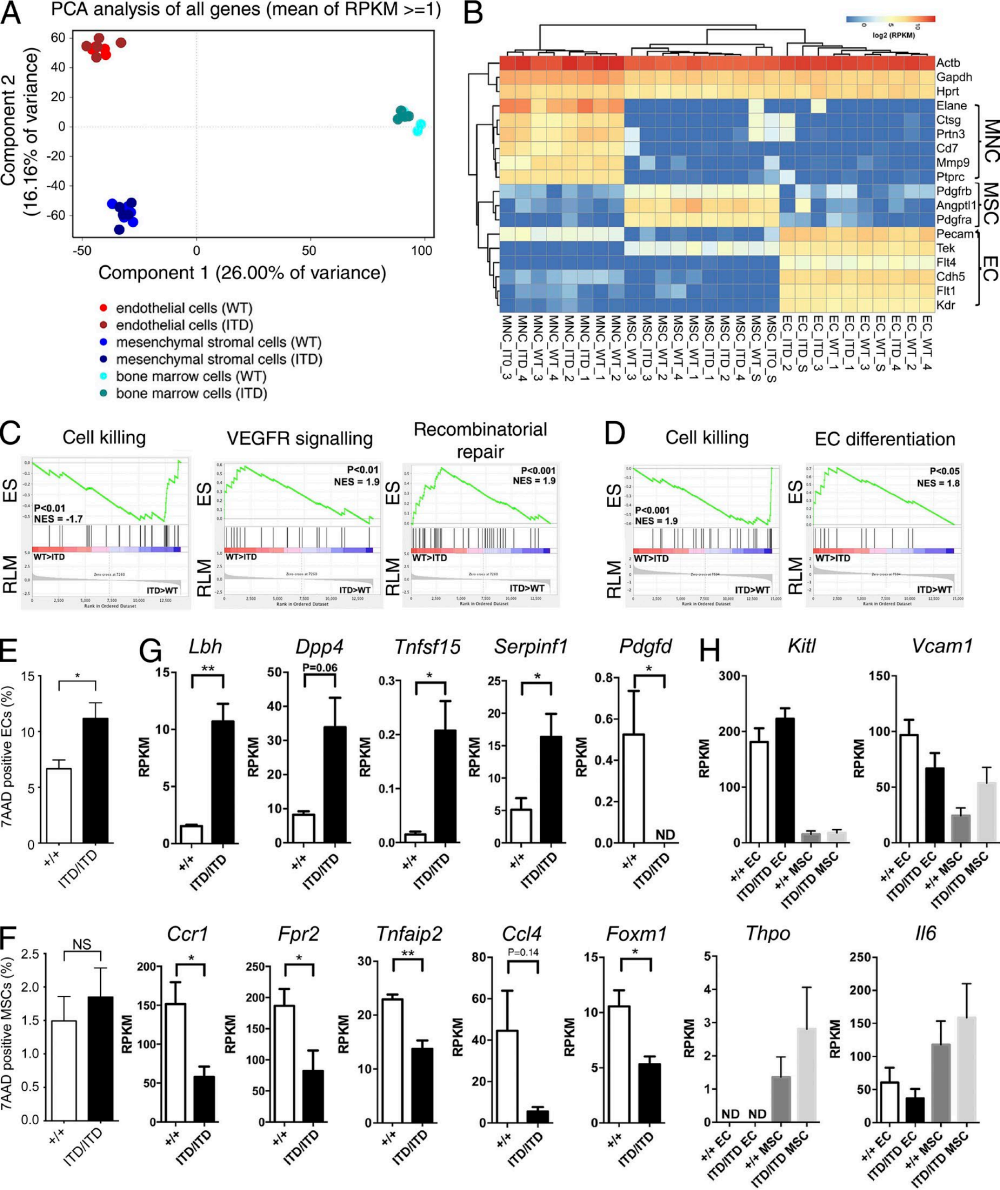
**Molecular features of niche elements in Flt3-ITD mice.** BM MNCs, ECs (CD45^−^Ter119^−^CD31^+^), and MSCs (CD45^−^Ter119^−^CD31^−^CD166^−^Sca1^+^) from *Flt3^+/+^* and *Flt3^ITD/ITD^* 8–10-wk-old mice (four to five mice per genotype) were subjected to global RNA sequencing. (A) Principal component analysis of MNCs, ECs, and MSCs from *Flt3^+/+^* and *Flt3^ITD/ITD^* mice. Each dot represents the indicated cell population from one mouse. (B) Expression of hematopoietic, endothelial, and MSC-associated genes in purified populations of MNCs, ECs, and MSCs from *Flt3^+/+^* and *Flt3^ITD/ITD^* 8–10-wk-old mice (four to five mice per genotype). (C) GSEA comparing ECs from *Flt3^+/+^* and *Flt3^ITD/ITD^* mice for genes involved in regulation of cell killing and regulation of vascular endothelial growth factor receptor signaling pathway and DNA repair (five mice per genotype). (D) GSEA genes comparing MSCs from *Flt3^+/+^* and *Flt3^ITD/ITD^* mice for genes involved in regulation of cell killing and regulation of endothelial cell differentiation (five mice per genotype). (E and F) Frequency of 7AAD-positive ECs (E) and MSCs (F) in the BLC of 8–10-wk-old *Flt3^+/+^* and *Flt3^ITD/ITD^* mice (six and seven mice per genotype, respectively). (G) Aberrant expression of angiogenesis-associated genes in MNCs from *Flt3^ITD/ITD^* mice (four mice per genotype). (H) Expression of genes implicated in extrinsic regulation of HSCs in EC and MSC from *Flt3^+/+^* and *Flt3^ITD/ITD^* mice (five mice per genotype). Data are shown as mean (SEM) values. *, P < 0.05; **, P < 0.01 by *t* test. ND, not detected; NS, not significant.

We next performed RNA sequencing of purified ECs, MSCs, and total BM mononuclear cells (MNCs) from BLCs of *Flt3^+/+^* and *Flt3^ITD/ITD^* mice (Nakamura et al., 2010). Principle component analysis confirmed that each of the three cell populations tightly clustered in *Flt3^+/+^* and *Flt3^ITD/ITD^* mice (Fig. 6 A) and that lineage-specific genes were selectively expressed (Fig. 6 B). However, differential gene expression between *Flt3^+/+^* and *Flt3^ITD/ITD^* ECs, MSCs, and MNCs was detected for 392, 308, and 428 genes, respectively. In keeping with the reduced numbers of ECs and MSCs in *Flt3^ITD/ITD^* mice, gene set enrichment analysis (GSEA) showed increased expression of genes associated with cell killing in *Flt3^ITD/ITD^* ECs; reduced expression of vascular endothelial growth factor receptor signaling pathway genes, a key regulator of angiogenesis (Breier, 2000); and impaired DNA repair (Fig. 6 C). Furthermore, GSEA demonstrated similarly enhanced expression of pathways involved in cell killing in *Flt3^ITD/ITD^* MSCs and impaired regulation of EC differentiation (Fig. 6 D). In further support of increased cell death in ECs, frequency of 7AAD-positive cells was increased in ECs and MSCs from *Flt3^ITD/ITD^* mice (Fig. 6, E and F), although the increase did not reach statistical significance for MSCs. In keeping with these findings, analysis of specific genes involved in regulation of angiogenesis in MNCs (Fig. 6 G) revealed up-regulation of several angiogenesis-inhibiting genes in *Flt3^ITD/ITD^* mice (*Lbh, Dpp4, Tnfsf15*, and *Serpinf1)* and down-regulation of angiogenesis-promoting factors (*Pdgfd*, *Ccl4, Ccr1, Fpr2, Tnfaip2*, and *Foxm1*). In contrast, expression of key steady-state extrinsic regulators of HSCs (*Kitl, Vcam1, Thpo*, and *Il6*) was not significantly affected in ECs and MSCs (Fig. 6 H).

**Figure 7.**
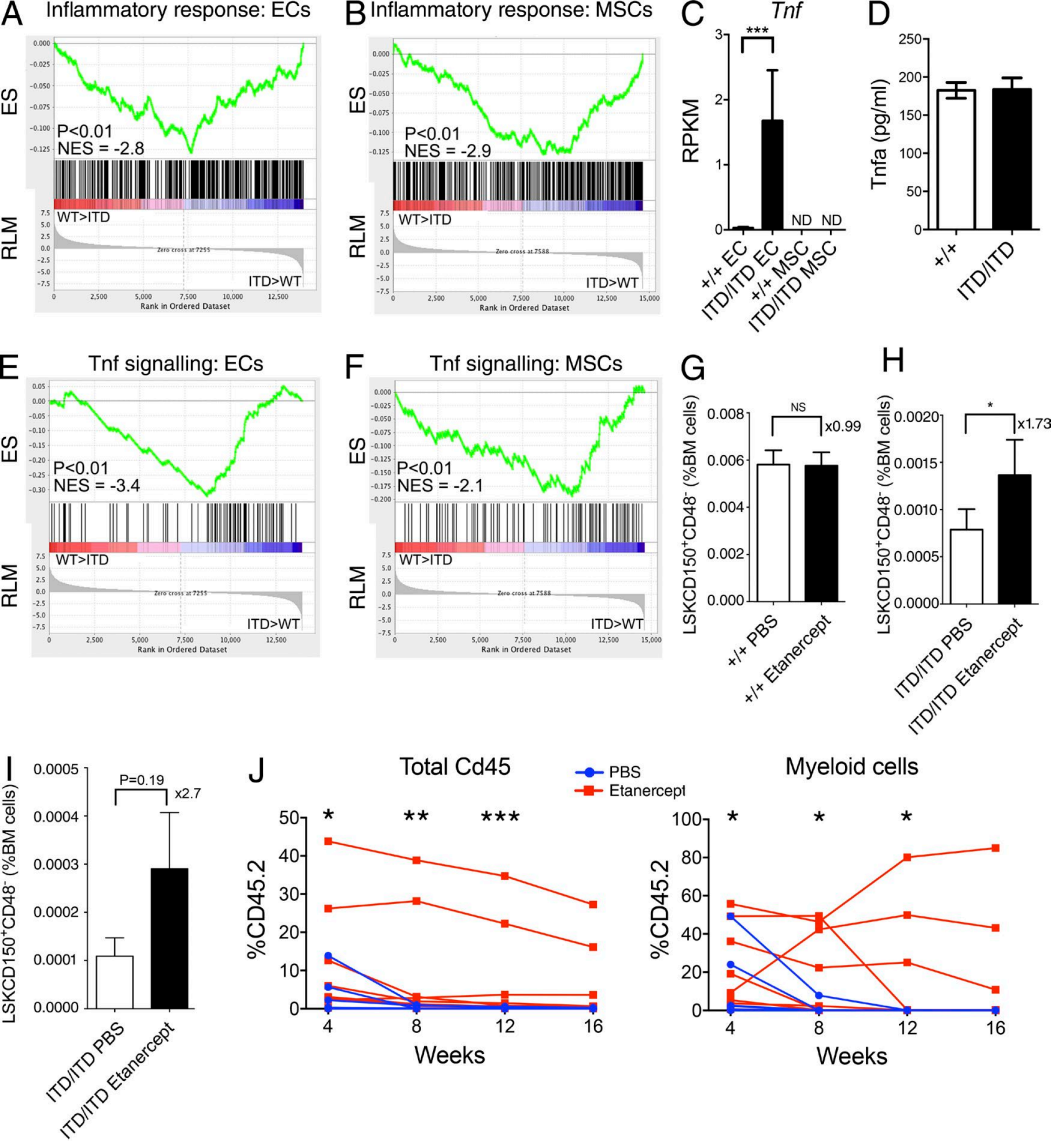
**Aberrant inflammation and TNF signaling cause HSC suppression in Flt3-ITD mice.** (A and B) GSEA comparing ECs (A) and MSCs (B) in *Flt3^+/+^* and *Flt3^ITD/ITD^* mice for inflammatory response–associated gene expression (five mice per genotype in two experiments). (C) Increased expression of *Tnf* in ECs from *Flt3^ITD/ITD^* mice (five mice per genotype). Adjusted p-value from edgeR analysis is shown. (D) Serum TNF-α levels in *Flt3^+/+^* and *Flt3^ITD/ITD^* 8–10-wk-old mice (two to three mice per genotype). (E and F) GSEA comparing ECs (E) and MSCs (F) in *Flt3^+/+^* and *Flt3^ITD/ITD^* mice for TNF signaling–associated gene expression (five mice per genotype). (G and H) Impact of 3 wk of etanercept treatment on numbers (percentage of total BM cells) of phenotypic (LSKCD150^+^CD48^−^) HSCs in *Flt3^+/+^* (G) and *Flt3^ITD/ITD^* (H) mice (*n* = 6–7 mice per group in two independent experiments). (I) Impact of etanercept treatment (compared with PBS treatment) on numbers of phenotypic competitor HSCs (CD45.1^+^LSKCD150^+^CD48^−^) in recipient mice 12 wk after transplantation of 5 × 10^5^ CD45.2 E15 FL cells (Flt3^ITD/ITD^) into CD45.1 WT recipients with 10^6^ WT CD45.1 BM competitor cells. Data are shown as mean (SEM) values (*n* = 6 mice per treatment group, two independent experiments). (J) Serial analysis of percentage CD45.2 chimerism in peripheral blood of recipient mice after transplantation of 500,000 unfractionated CD45.2 BM cells from *Flt3^ITD/ITD^* mice after 3 wk of treatment with PBS or etanercept (*n* = 9 mice per treatment group in two experiments). WT CD45.1 competitor cells (*n* = 200,000) were cotransplanted. Data are shown for total PBMCs (left) and Mac1^+^Gr1^−^ myeloid cells (right). P-values were generated using Mann–Whitney test to compare percentage of CD45.2 across all replicates for each time point. *, P < 0.05; **, P < 0.01; ***, P < 0.001. ND, not detected; NS, not significant.

We next performed MetaCore analysis of the differentially expressed genes in MNCs, ECs, and MSCs. Strikingly, inflammation pathways were disrupted in all three cell types (Table S1). Furthermore, GSEA showed up-regulation of gene expression associated with inflammatory response in both ECs (Fig. 7 A) and MSCs (Fig. 7 B). Of particular note, expression of the proinflammatory cytokine *Tnf-α* (*Tnf*) was up-regulated in ECs (Fig. 7 C). Notably, serum levels of TNF were not increased in *Flt3^ITD/ITD^* mice (Fig. 7 D), suggesting that the up-regulation of *Tnf* in BM endothelial cells might result in a local effect. In keeping with increased TNF signaling in the BM microenvironment, GSEA showed up-regulation of gene expression associated with TNF signaling in both ECs (Fig. 7 E) and MSCs (Fig. 7 F). Inflammation is known to be an important suppressor of HSC function (King and Goodell, 2011; Schuettpelz and Link, 2013), including TNF, which is notably one of the few known cell-extrinsic suppressors of HSCs (Pronk et al., 2011). We reasoned that the increased TNF expression in the BM of *Flt3^ITD/ITD^* mice might account for some of the suppression of HSCs observed. We therefore treated *Flt3^+/+^* and *Flt3^ITD/ITD^* mice with the clinically approved TNF inhibitor etanercept for 3 wk. Whereas the numbers of phenotypic HSCs were not affected in *Flt3^+/+^* mice (Fig. 7 G), they were significantly increased in *Flt3^ITD/ITD^* mice (Fig. 7 H). CD45.1 competitor HSCs analyzed 12 wk after transplantation of 5 × 10^5^ CD45.2 E15 *Flt3^ITD/ITD^* FL cells with 10^6^ WT CD45.1 BM competitor cells showed a similar increase after etanercept treatment, although the difference was not statistically significant (Fig. 7 I). Transplantation of BM cells from PBS or etanercept-treated *Flt3^ITD/ITD^* mice showed the expected lack of long-term engraftment in all PBS-treated mice (*n* = 9; Fig. 7 J). However, three of nine mice treated with etanercept showed sustained engraftment to 16 wk with no evidence of a loss of engraftment that would be anticipated if there was a cell-intrinsic deleterious effect of Flt3-ITD on HSCs (Fig. 7 J). This finding is in marked contrast to the lack of sustained engraftment in 24 recipient mice transplanted with 500,000 BM cells from non–etanercept-treated *Flt3^ITD/ITD^* mice (P < 0.01). Collectively, these data demonstrate that *Flt3^ITD/ITD^*-driven myeloproliferation creates a proinflammatory BM microenvironment, including overexpression of *Tnf* in BM niche cells, that causes cell-extrinsic suppression of non–Flt3-expressing HSCs.

## Discussion

In the present study, we demonstrated a progressive and gene dosage–dependent severe suppression of phenotypically and functionally defined HSCs in Flt3-ITD knock-in mice, in keeping with previous work (Chu et al., 2012). Several previous studies, including *Flt3*-Cre fate mapping, concluded that Flt3 protein as well as mRNA expression in normal mouse hematopoiesis initiates in MPPs rather than bona fide HSCs (Adolfsson et al., 2001; Christensen and Weissman, 2001; Buza-Vidas et al., 2009, 2011; Boyer et al., 2011). This would suggest that the mechanism through which Flt3-ITDs affect the HSC reservoir is unlikely to be intrinsic to HSCs. However, a recent study implied a previously unrecognized intrinsic impact of Flt3 in HSC homeostasis by concluding that the FLT3-ITD–induced HSC suppression is not extrinsically, but rather HSC-intrinsically, mediated in Flt3-ITD knock-in mice (Chu et al., 2012). The distinction between a cell-intrinsic or -extrinsic effect of Flt3-ITD on the HSC compartment is of considerable importance for understanding the role of Flt3 in normal hematopoiesis and the impact of Flt3-ITDs on HSCs in hematologic malignancies. We therefore investigated the possibility of HSC-intrinsic and -extrinsic impacts of Flt3-ITD through a combination of single-cell interrogation of *Flt3* expression in the phenotypically defined HSC compartment, functional assays, and examination of key components of the HSC niche.

If an HSC-intrinsic impact of Flt3-ITDs on HSCs were to explain the severe reduction in HSC numbers in Flt3-ITD mice, then the expectation would be that the Flt3 receptor is expressed in the large majority of HSCs, because the mutation cannot exert an intrinsic impact on HSCs when it is not expressed. Herein, using a highly sensitive nanofluidic approach to interrogate *Flt3* expression at the single-cell level, we found that only a small fraction of LSKCD150^+^48^−^ cells expressed *Flt3* mRNA, and at very low levels. Furthermore, this infrequent *Flt3* expression was inversely correlated with expression of several HSC-associated genes, including *Vwf*, a highly specific marker of platelet-biased HSCs (Sanjuan-Pla et al., 2013). Furthermore, single-cell gene expression analysis of Flt3-ITD mice suggested that the phenotypic HSC compartment is composed primarily of progenitor cells, with a selective depletion of *Flt3* mRNA–negative bona fide HSCs, which represent only a small minority of cells within the LSKCD150^+^48^−^ population in Flt3-ITD mice. Indeed, it has been widely recognized that LSKCD150^+^48^−^ cells (and any other phenotypically defined HSC population) also contain a substantial fraction of non-HSCs, and that the proportion of such contaminating non-HSCs within the phenotypically defined HSC compartment might be increased in genetically modified mice (Purton and Scadden, 2007), in particular if the fraction of bona fide HSCs is selectively depleted, such as described here in Flt3-ITD mice. This selective loss of *Flt3* mRNA–negative and Vwf-positive LSKCD150^+^48^−^ cells in Flt3-ITD mice suggests that the LSKCD150^+^48^−^ compartment in Flt3-ITD mice contains a much lower proportion of bona fide HSCs than in WT mice. This was further supported by the dramatic loss of long-term reconstituting activity of BM cells from Flt3-ITD mice as established through competitive transplantation experiments. These findings highlight the clear advantage of single-cell analysis to reveal cellular heterogeneity in phenotypically defined cell types that is not apparent at the cell population level (Wills and Mead, 2015), a finding that has potentially important implications for the analysis of other disease models.

It is not possible to exclude the possibility that some *Flt3* mRNA might be expressed below the limit of detection of the assays; however, our single-cell gene expression analysis did not provide any support for a HSC-intrinsic suppression of bona fide HSCs in Flt3-ITD mice, and we therefore explored the possibility of a cell-extrinsic mechanism. Although a competitive reconstitution assay (Purton and Scadden, 2007) is the only manner in which one can conclusively establish whether an observed reduction in HSC numbers is intrinsically or extrinsically mediated, reliable interpretation of the results depends entirely on a comparable number of genuine HSCs being transplanted from the genetically modified and control WT mice. The extent of contaminating non-HSCs might be altered extensively in genetically modified mice, in particular if such non-HSCs are expanded compared with the WT setting or if the true HSCs are selectively depleted, as specifically demonstrated to be the case in Flt3-ITD mice through single-cell gene expression analysis. Thus, when HSC numbers as well as the HSC functional readout are already severely affected by a mutation such as *Flt3-ITD*, transplantation of equal numbers of functional HSCs is difficult if not impossible to achieve. Failure to take this heterogeneity into account can lead to false assumptions in the experimental design and incorrect interpretation of data. Consequently, transplantation of equal numbers of phenotypic HSCs will in reality result in transplantation of markedly lower numbers of functional HSCs from Flt3-ITD versus WT mice (Chu et al., 2012). In light of this, such a competitive assay must therefore be performed before a significant HSC phenotype has been acquired, through establishing BM chimeric mice either before the mutation is induced (requiring, unlike here and in the studies of Chu et al. [2012], a mouse model with an inducible mutation) or before the HSC phenotype has been acquired. In our case, this was possible by establishing BM chimeras with E15 FL cells, at which time the number of LSKCD150^+^48^−^ cells was unperturbed, likely because this is before the development of overt myeloproliferative disease in Flt3-ITD mice (Mead et al., 2013). Furthermore, FL HSCs are extremely robust at engraftment (Bowie et al., 2007), and it is a frequently observed phenomenon in the literature that FL HSCs can sustain high-level engraftment in mouse models associated with a postnatal HSC defect (Jones et al., 2015). Through this approach, we were able to provide definitive evidence of a marked cell-extrinsic effect of Flt3-ITDs on the HSC compartment, as WT competitor LSKCD150^+^48^−^ cells were dramatically reduced in the presence of competing Flt3-ITD–expressing cells, in fact more extensively than LSKCD150^+^48^−^ cells in the Flt3-ITD donor cells in the same mice, a finding in further support of LSKCD150^+^48^−^ cells in Flt3-ITD mice being relatively depleted of genuine HSCs and most compatible with the observed HSC suppression in Flt3-ITD mice being mediated fully cell-extrinsically. Importantly, if in fact HSCs were already somewhat reduced in the FL of Flt3-ITD mice, we would if anything have underestimated the level of cell-extrinsic suppression of HSCs in the competitive transplantation experiments performed. Distinguishing between a cell-extrinsic and -intrinsic impact of Flt3-ITD on HSCs is crucial, as it has extensive implications for the understanding of Flt3 as a key regulator of normal hematopoiesis and the impact and therapeutic targeting of FLT3-ITD mutations in hematologic malignancies. Our findings are in line with results from other models of hematologic malignancies (Schepers et al., 2013) and human disease (Colmone et al., 2008), supporting an extrinsic impact of some hematopoietic tumors on HSCs.

We also explored the possible mechanistic basis of the HSC-extrinsic suppression of HSCs in Flt3-ITD mice through analysis of key cellular components and molecular determinants of the HSC niche. We found a two- to fivefold reduction in the numbers of MSCs and ECs in the BM of Flt3-ITD mice, both niche cell populations suggested to be critical for the regulation of HSCs (Morrison and Scadden, 2014). Moreover, ECs and MSCs in Flt3-ITD mice demonstrated aberrant gene expression, consistent with increased apoptosis and impaired EC homeostasis. Analysis of total BM MNCs also revealed dysregulation of genes implicated in angiogenesis. In addition, mediators of inflammation known to be associated with suppression of HSCs, such as TNF-α (King and Goodell, 2011; Pronk et al., 2011), were overexpressed by ECs in Flt3-ITD mice. Furthermore, several inflammatory mediators are known to increase TNF expression in endothelial cells (Ranta et al., 1999; Imaizumi et al., 2000). Inflammation and TNF signaling have also been directly implicated in the disruption of BM microenvironment and hematopoiesis in patients with a range of myeloid malignancies (Chen et al., 2016; Koschmieder et al., 2016), including elevated TNF levels in patients with AML (Sanchez-Correa et al., 2013), correlating with high leukocyte count and resistant disease (Tsimberidou et al., 2008). We therefore treated Flt3-ITD mice with a clinically approved anti-TNF therapy (etanercept), which resulted in a partial rescue of the HSC phenotype in Flt3-ITD mice, as confirmed in functional, competitive HSC repopulation assays. Because etanercept treatment only partially rescued the HSC defect in Flt3-ITD mice, it is likely that other mechanisms of HSC repression, including other inflammatory mediators, might also play a role. Collectively, we observed both quantitative (reduction in numbers of ECs and MSCs) and qualitative (inflammation and TNF signaling) disruption of the BM microenvironment in Flt3-ITD mice, which likely underlie the observed extrinsic suppression of HSCs, in agreement with other studies implying that hematologic malignancies dysregulate HSC niches (Schepers et al., 2015).

Although genetically engineered mouse lines are imperfect model systems for human malignancies, our findings do raise several potentially highly relevant issues for the understanding of human AML biology. Because only HSCs are capable of long-term propagation of the Flt3-ITD clone (Chu et al., 2012), this is to our knowledge the first demonstration of a tumor model that is dependent on propagation by a stem cell compartment which carries the relevant DNA mutation but nevertheless does not express the mutant protein. Consequently, acquisition of the mutation by a HSC will have no intrinsic impact on the cell, exerting neither positive nor negative selection pressure. Although at a cell population level FLT3-ITD mutations are not easily detectable in preleukemic HSCs (Jan et al., 2012), it remains fully possible that rare HSCs might still carry the mutation, which upon subsequent differentiation and FLT3-ITD protein expression would confer a clonal advantage in a progenitor population, as here demonstrated for Flt3-expressing MPPs. Indeed, there is ample support for such FLT3-expressing progenitor populations being transformed to become leukemic stem cell populations in patients with AML (Goardon et al., 2011). The clinical use of small-molecule inhibitors of FLT3 signaling has in general been disappointing in AML (Kayser and Levis, 2014), with frequent acquisition of resistance causing mutations in patients demonstrating an initial response (Smith et al., 2012). This suggests that inhibitor-insensitive FLT3-ITD mutated cells, potentially because they do not express the mutant protein, remain in responding patients, acting as a reservoir for the acquisition of additional mutations (Smith et al., 2012). Given the lack of expression of the mutant protein, such mutant HSCs would not have a competitive advantage at the HSC level, but would nevertheless be very relevant for the initiation of AML and propagation of relapse, because they would not be targeted by FLT3 inhibitors. Studies of leukemic stem cell populations would be insufficiently sensitive to detect such extremely rare cells, and further studies on larger patient cohorts to explore this possibility with single-cell resolution are therefore warranted.

## Materials and methods

### Animals

All mice were bred and maintained in accordance with UK Home Office regulations, and experiments were conducted after approval by the University of Oxford Animal Welfare and Ethical Review Body (project license 30/3103). Flt3-ITD knock-in mice on C57BL/6 background have been previously described (Lee et al., 2007; Kharazi et al., 2011). Homozygous (*Flt3^ITD/ITD^*) and heterozygous (*Flt3^ITD/+^*) Flt3-ITD mice were studied as specified. Vwf-EGFP mice have been previously described (Sanjuan-Pla et al., 2013) and were on a C57BL/6 background. Mice were analyzed with littermate controls of the appropriate genotype when available and as specified.

### FACS

Specific details of mouse antibodies and viability dyes used for flow cytometry analysis are shown in Table S2. All antibodies were used at predetermined (titrated) optimized concentrations. Cell acquisition and analysis were performed on a four-laser LSRII (BD) using FlowJo software (Tree Star). Myeloid progenitor populations were identified as previously described (Kharazi et al., 2011). Cell sorting was done on a BD FACSAria II cell sorter. Cells used in cell sorting experiments were not enriched and were stained after initial Fc-block incubation. Gates were set using a combination of fluorescence-minus-one controls and also populations that are known to be negative for the antigen.

### Competitive transplantation assay

Congenic CD45.1 and CD45.2 WT C57BL/6 mice were used as controls and recipients in transplantation experiments. Adult (8–10-wk-old mice) BM transplantation was performed using 500,000 donor CD45.2 BM cells, or purified cell populations as indicated, from *Flt3^+/+^* or *Flt3^ITD/ITD^* mice along with 200,000 CD45.1 WT competitor cells. FL transplantations used 500,000 donor *Flt3^+/+^* or *Flt3^ITD/ITD^* CD45.2 FL cells with 1,000,000 CD45.1 WT BM competitor cells. Cells were injected into tail veins of lethally irradiated (900 cGy) recipient CD45.1 mice. Flow cytometric analysis of CD45.1 and CD45.2 reconstitution and donor-derived lineage and progenitor reconstitution were assessed 12 wk after transplantation as indicated and previously reported (Kharazi et al., 2011).

For etanercept treatment, 8-wk-old WT C57BL/6J and *Flt3^ITD/ITD^* mice were injected intraperitoneally with PBS or etanercept (Pfizer) at a dose of 10 mg/kg body weight three times a week for 3 wk before BM cells were collected for transplantation. For etanercept treatment of transplanted mice, 500,000 unfractionated WT or Flt3-ITD E15 FL cells (CD45.2 allotype) were intravenously injected along with 1,000,000 WT unfractionated CD45.1 BM cells into lethally irradiated CD45.1 recipients (900 cGy, split dose). Transplanted mice were injected intraperitoneally with PBS or etanercept (Pfizer) at a dose of 10 mg/kg body weight three times a week from 6 wk posttransplantation until the final analysis. Peripheral blood reconstitution was monitored at 4, 8, and 12 wk after transplantation. 12 wk after transplantation, phenotypic analysis of BM HSCs was performed.

### In vitro HSC survival assay

Single LSKCD150^+^48^−^ HSC-enriched cells or LSKFlt3^high^ LMPPs were preincubated in 10 µl Stemspan SFEM (STEMCELL Technologies) supplemented with 100 ng/ml mouse SCF, 100 ng/ml human FLT3L, or no cytokines as indicated for 5 d. Next, an additional 10 µl medium containing a highly proliferative cytokine cocktail was added into each well. Final concentrations of cytokines for all cells were 50 ng/ml mSCF, 50 ng/ml FLT3L, 5 ng/ml human thrombopoietin, 5 ng/ml murine IL-3, 10 ng/ml murine GM-CSF, and 10 ng/ml human G-CSF. After an additional 10 d of incubation at 37°C, 98% humidity, and 5% CO_2_, wells were analyzed under an inverted light microscope for cells that had undergone at least one cell division.

### Preparation of single-cell suspension of stromal cells

For preparation of BLCs, femurs and tibias were harvested, cleaned of excess muscle tissue, and placed into PBS/5% FCS. Bones were crushed in a mortar, and BM was removed for separate processing of central BM. The bones were fragmented with scissors and transferred to a 15-ml tube (Falcon). Bones were spun down, and excess PBS/FCS was removed. Cells were isolated by incubation at 37°C with type I collagenase (3 mg/ml; Worthington Biochemical Corporation) for 45 min with rotation in DMEM with 10% FCS. The cell suspension was collected by passing through a 70-µm cell strainer. Collagenase was then added for a further 45 min. Cells were collected and washed in PBS/5% FCS/2 mM EDTA to reduce clumping.

For analysis of central BM stromal cells, BM cells were incubated at 37°C with type IV collagenase (2 mg/ml; Worthington Biochemical Corporation) for 20 min with rotation in DMEM with 10% FCS, and RBCs were lysed with ammonium chloride solution (STEMCELL Technologies) at room temperature for 10 min. Subsequently, CD45^+^ cells were depleted with anti-CD45 MicroBeads (Miltenyi Biotec). Cells were collected and resuspended in PBS/5% FCS/2 mM EDTA for FACS analysis.

### Multiplex quantitative real-time PCR analysis of single cells and cell populations

For cDNA synthesis and preamplification of target genes, CellsDirect One-Step qRT-PCR kit (Invitrogen) was used. Cells were sorted directly into PCR plates or 0.2-ml PCR tubes containing 2.5 µl gene-specific 0.2× TaqMan gene expression assays (Applied Biosystems), 5 µl CellsDirect 2× Reaction mix (Invitrogen), 1.2 µl CellsDirect RT/Taq mix, 1.2 µl TE buffer, and 0.1 µl SUPERase•In RNase Inhibitor (Ambion) to a total volume of 10 µl. For single cells, the reaction volume was reduced to 5 µl. Conditions for reverse transcription and target gene amplification were 15 min at 50°C; 2 min at 95°C; and 22 cycles of 95°C for 15 s and 60°C for 4 min. Preamplified products were diluted 1:5 in TE buffer and analyzed on Dynamic Array (Fluidigm) using the following PCR cycling conditions: 95°C for 10 min and 40 cycles of 95°C for 15 s and 60°C for 60 s. Data were analyzed using the ΔCt method; results were normalized to *Hprt* expression for cell populations and *Gapdh* expression for single-cell analysis and expressed as relative expression level. Single cells not expressing *B2m*, *Hprt*, or *Gapdh* were excluded from the analysis. For t-SNE analysis, single-cell expression data were aggregated, and genes with zero variance were excluded from the analysis. The Ct value range was truncated to a maximum value of 40, in excess of the maximum recorded Ct. The t-SNE (van der Maaten and Hinton, 2008; van der Maaten, 2014) transformation was performed in Matlab (v. R2015a, 8.5.0.297613) using all 96 murine genes included in the gene expression analysis, and transformed data were plotted in R. Cells were hierarchically clustered on the basis of their expression profiles. The resulting dendrogram was used to identify two major clusters of cells. The deviation of the frequency of each population from a uniform partitioning between the two clusters was assessed by means of binomial tests. Details of TaqMan assays used are shown in Table S3.

### Nanofluidic cell capture and single-cell gene expression analysis

Cell capture and target preamplification were performed using the C1 system (Fluidigm) according to the manufacturer’s instructions, using forward, 5′-GTGGCCAAAGGCATGGAATT-3′, and reverse, 5′-TGGGTGACCAACACATTCCT-3′ primers (Fluidigm DELTAgene assays). Real-time PCR was performed on preamplified material from single cells using the BioMark 96.96 Dynamic Array platform (Fluidigm).

### RNA sequencing

Samples for RNA sequencing were prepared using the SMARTer Ultra Low RNA kit for Illumina Sequencing (Takara Bio Inc.) according to the manufacturer’s instructions as previously described (Böiers et al., 2013; Woll et al., 2014). In brief, cDNA libraries were prepared from 100 (EC and MSC) or 200 (MNC) cells, with four to five biological replicates from each genotype. Cells were sorted directly into lysis buffer supplemented with RNase Inhibitor (Takara Bio Inc.). cDNA libraries were prepared according to manufacturer instructions with 15 cycles of amplification. Amplified cDNA libraries validated with a distinct peak spanning 400–9,000 bp in size as measured by High Sensitivity DNA kit (Agilent Technologies) on Agilent 2100 Bioanalyzer were further processed for RNA sequencing. Libraries were prepared for Illumina sequencing using the Nextera XT DNA Sample Preparation kit (Illumina) using the manufacturer’s recommended protocol. The size of the amplified fragments was confirmed by Agilent DNA1000 kit (Agilent Technologies). Four to five samples with different indexes were pooled per lane and sequenced on a HiSeq2000 (Illumina) generating single-end, 51-bp reads. After quality control analysis, reads were aligned using Bowtie (Langmead et al., 2009) against the murine transcriptome (mouse NCBI build38 [mm10] UCSC transcripts). Non–uniquely mapped reads were discarded. Expression levels of all genes were quantified using RSEM with default parameters (Li and Dewey, 2011). The estimated read count for each gene generated by RSEM was used for statistical calculation of global differential gene expression between specified populations using DESeq2 (Anders and Huber, 2010) and edgeR (Robinson et al., 2010) packages. Genes were considered differentially expressed between populations at an adjusted p-value <0.1 from either DESeq2 or edgeR and log_2_ fold change of reads per kilobase of transcript per million mapped reads (RPKM) values ≥1. The RPKM values estimated by RSEM were used when investigating the expression of specific genes. Hierarchical clustering and principal components analysis were performed in R. The *pheatmap* function was used to generate the heatmap and the *prcomp* function was used for PCA analysis on the log-transformed RPKM values. GSEA analysis (Subramanian et al., 2005) was performed using GSEA software (http://www.broadinstitute.org/gsea). Pathways enrichment analysis was performed using MetaCore software (Thomson Reuters). Data files have been uploaded to GEO database (accession no. GSE63206).

### Immunohistochemistry

Femoral bones were cryoembedded in OCT compound and sectioned at 8-µm thickness. Sections were fixed with methanol for 15 min and blocked with a protein blocker (Dako). Specimens were then incubated with goat anti–VE-cadherin antibody (R&D Systems) followed by donkey anti–goat IgG antibody conjugated to Alexa Fluor 488 (Molecular Probes) and nuclear staining. Images were acquired with a confocal laser-scanning microscope (FV1000; Olympus). Blood vessel perimeters were measured using ImageJ (NIH).

### ELISA

Mouse serum samples were collected from peripheral blood clotted for 2 h at room temperature by centrifuging for 20 min at 2,000 *g*. Samples were assessed by murine TNF ELISA kits (Abcam) according to the manufacturer’s instructions. Optical density measurements were determined using a microplate reader (SpectraMax M2e; Molecular Devices) set to 450 nm with wavelength correction set to 570 nm.

### Statistical analysis

Unless otherwise indicated, the statistical significance of differences between samples was determined using a Student’s *t* test for normally distributed data and Mann–Whitney test for non–normally distributed data.

### Online supplemental material

Table S1 shows metacore analysis of ECs and MSCs. Table S2 lists the antibodies used. Table S3 lists the TaqMan assays used.

## Supplementary Material

Table S1 (Excel file)

Table S2 (Excel file)

Table S3 (Excel file)
